# 16S rRNA Gene Amplicon Sequencing and Functional Profiling of Rhizosphere Bacteria in Date Palms Reveal Diverse Potential Plant Growth–Promoting Traits

**DOI:** 10.1155/ijm/9216273

**Published:** 2026-06-23

**Authors:** Sultan M. Alsharif, Mohamed Ismaeil, Hibah M. Albasri, Wael S. El-Sayed

**Affiliations:** ^1^ Department of Biology, College of Science, Taibah University, Madinah, Saudi Arabia, taibahu.edu.sa; ^2^ Microbiology Department, Faculty of Science, Ain Shams University, Cairo, Egypt, asu.edu.eg; ^3^ School of Biotechnology, Badr University in Cairo (BUC), Cairo, Egypt

## Abstract

The rhizosphere of date palm trees serves as a reservoir for plant growth–promoting bacteria (PGPB). To characterize the microbial communities and associated PGP traits within these rhizospheres, Illumina amplicon sequencing of 16S rRNA genes and Phylogenetic Investigation of Communities by Reconstruction of Unobserved States (PICRUSt) analysis were conducted on samples from four cultivars (Ajwa, Helwa, Rabeaa, and Ruthana) collected in Madinah, Saudi Arabia. Proteobacteria were the dominant bacterial group in most samples, except for Rabeaa, where *Actinobacteria* were more prevalent. The unclassified PAC002137_g genus was most abundant overall. A diverse array of PGPB genera, including *Bacillus*, *Arthrobacter*, *Lysobacter*, *Paenibacillus*, and *Mesorhizobium*, was identified. PICRUSt analysis revealed a plethora of potential PGP genes, with those involved in phosphate and sulfate transport, iron acquisition, and redox processes being particularly abundant. These findings suggest that the date palm rhizosphere harbors a rich microbial community capable of promoting plant growth through various mechanisms. Further investigations into the specific roles of these PGPBs could lead to the development of sustainable agricultural practices for date palm cultivation.

## 1. Introduction

Date palm (*Phoenix dactylifera* L.), a dioecious, perennial monocotyledon, is a resilient crop adapted to arid and semiarid environments [1]. Native to the Arabian Peninsula, it has been cultivated for millennia and holds significant cultural and economic importance in Arabian countries [2]. With optimal growth between 17.5°C and 27.5°C and tolerance to high temperatures and soil salinity, date palm exhibits unique biological traits that require specialized cultivation practices [3]. It is a primary economic crop in regions such as Saudi Arabia and contributes significantly to agricultural production and socioeconomic development. The genetic diversity of date palms, represented by numerous cultivars, offers the potential for crop improvement and adaptation to changing environmental conditions [4–10].

Madinah, a leading date producer in Saudi Arabia, is known for its date cultivars with high nutritional value [11, 12]. Notably, Madinah is the sole source of the Ajwa cultivar (*P. dactylifera* L. cv. Ajwa), which is resistant to cultivation elsewhere [13]. Other historically significant cultivars include Rabeaa, Ruthana, and Helwa [14].

Date palm fruits are rich in sugars, amino acids, vitamins, fatty acids, and minerals and are nutritional staples with documented antioxidant, antimutagenic, anti‐inflammatory, antibacterial, and antifungal properties [12, 13, 15, 16].

Date palm exhibits extensive genetic diversity, with nearly 5000 cultivars reported globally, often accompanied by synonymy and homonymy [17]. Previous studies have characterized diverse date palm varieties across different regions [18–22]. Saudi Arabia alone boasts more than 350 cultivars, which are distinguished by their morphological, nutritional, and commercial attributes [23]. Prominent commercial cultivars in Saudi Arabia include Rushoda, Nabtatali, Khala, Dekhaini, Ajwa, Khashram, Khodry, Nabootsaif, Majhool, Sullaj, Shalabi, Shaishee, Sukkari, Segae, Ruthana, Barni Al‐Madina, Sabaka, Luban, Rabeaa, Hilali, Helwa, Safawi, Ruthana Alsharag, Anbara, Mutwah, Meneifi, Mabroom, Sefri, and Deglet Noorin [24]. Notably, Ajwa, Rabeaa, Helwa, and Ruthana are renowned cultivars endemic to the Madinah Region. Date palm cultivars from the Madinah Region—Ajwa, Rabeaa, Ruthana, and Helwa—exhibit distinct nutritional profiles and potential health benefits [25–31].

Overall, soil microorganisms play a pivotal role in shaping ecosystem processes and increasing ecological resilience and complexity [32]. Research on the rhizosphere of date palms has revealed a wide range of beneficial interactions between date palms and plant growth–promoting (PGP) microbes, such as stimulating shoot and root growth [33], increasing tolerance to abiotic stresses [34], and suppressing pathogenic fungi [35]. Date palm sustainability can be enhanced through the targeted use of native plant–associated microbiomes, especially desert‐adapted microorganisms, which help plants cope with abiotic stresses such as drought and salinity. These microbes have been shown to improve seedling survival [36], increase nutrient uptake, increase resistance to pathogens [37], and support metabolic functions. As a result, harnessing beneficial microbes from arid environments presents a promising biotechnological approach to support and restore agriculture in desert regions, particularly in oasis‐based date palm cultivation [38].

The date palm rhizosphere harbors a diverse microbial community, primarily composed of bacteria and fungi, which is influenced by a complex interplay of biotic and abiotic factors, including cultivar, soil composition, geographical location, plant community structure, and agricultural practices [39–41]. In response to the extreme aridity and high temperatures of the Middle East and North Africa, date palm relies on its associated microbiota to facilitate growth and resilience under adverse conditions [42–44]. Recent advancements in omics technologies, particularly next‐generation sequencing, have enabled comprehensive characterization of the date palm rhizosphere microbiome, offering new avenues for developing sustainable agricultural practices centered on biofertilization and promotion of plant growth [45–48]. The ability of the rhizosphere to serve as a reservoir of indigenous microbial isolates that are well adapted to local environmental conditions has spurred increased research into the microbial ecology of date palm root systems [49].

The rhizosphere microbiomes of date palm cultivars exhibit significant diversity and variation. Comparative metagenomic analysis of Sukkari and Khalas date palms revealed distinct bacterial communities [50]. Actinobacteria isolated from date palm rhizospheres have demonstrated the potential to increase soil fertility and fruit quality; increase the phytochemical content; and induce antioxidant, antimicrobial, and anticancer properties [51]. Diverse actinomycete taxa, including *Streptomyces*, have been identified in Algerian date palm rhizospheres and exhibit antimicrobial activities [52]. Additionally, *Streptomyces* isolates from various date palm cultivars have demonstrated mycotoxin reduction capabilities [53]. These findings underscore the importance of rhizosphere microorganisms in promoting date palm health and productivity.

The composition of the microbial communities in both the rhizosphere and bulk soil of Tunisian date palms revealed a diverse ecosystem encompassing 22 bacterial phyla, with Gammaproteobacteria and Alphaproteobacteria dominating the root system [54]. A broader survey across seven Tunisian oases revealed a complex rhizosphere microbiome harboring plant growth–promoting bacteria (PGPB) crucial for plant homeostasis [55]. The potential of rhizosphere bacteria to alleviate abiotic stress was demonstrated by *Pseudomonas fluorescens* 002, which was isolated from Algerian date palms. This strain significantly increased maize root growth under saline and acidic conditions, highlighting its potential as a biotechnological tool for improving crop performance in challenging environments [56].

The rhizosphere of date palms harbors diverse microbial communities that influence plant growth and stress tolerance. The salinity of irrigation water significantly affects bacterial community composition, with specific taxa associated with salinity tolerance [57]. Plant growth–promoting rhizobacteria (PGPR) isolated from date palm rhizospheres exhibit potential for nutrient solubilization, hormone production, and the biocontrol of pathogens [58]. Additionally, endophytic bacteria, such as *Enterobacter cloacae*, have demonstrated PGP abilities [59].

Understanding the complex interactions among date palms, their associated microbiota, and environmental factors is crucial for developing sustainable management strategies.

This study investigated the bacterial community composition of date palm rhizospheres from the Madinah Region, which represents a knowledge gap in the literature. With a focus on the predominant cultivars (Ajwa, Rabeaa, Helwa, and Ruthana), we employed 16S rRNA amplicon sequencing to characterize the bacterial community structure and identify potential PGPB. Additionally, the Phylogenetic Investigation of Communities by Reconstruction of Unobserved States (PICRUSt) analysis was used to predict functional gene content and identify potential biomarkers associated with PGPB traits.

## 2. Materials and Methods

### 2.1. Sample Collection

**Figure 1:**
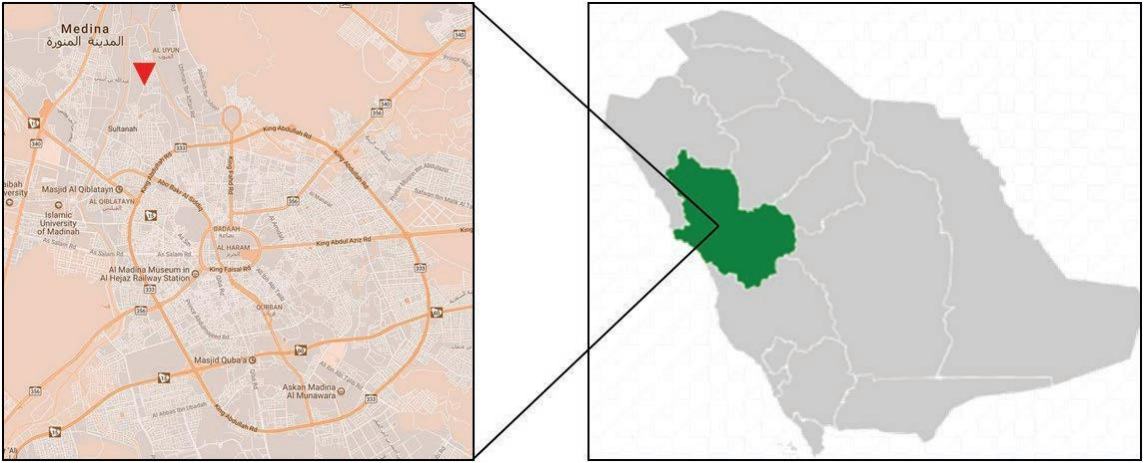
Geographical map showing the sampling site in the northern part of Madinah, Saudi Arabia.

Soil samples were collected in August 2020 from the rhizosphere of four date palm cultivars (Ajwa, Helwa, Rabeaa, and Ruthana) cultivated in a commercial orchard located in northern Madinah, Saudi Arabia (Figure 1). The rhizosphere soil samples employed in this study were designated AJW, HEL, RAB, and ROT and refer to the Ajwa, Helwa, Rabeaa, and Ruthana date palm cultivars, respectively. The orchard was managed under standard agricultural practices, including flood irrigation with groundwater (mean salinity: 1100 ppm) and organic fertilization. A total of eight samples were collected in this study, with two samples taken for each cultivar. Each sample consisted of a composite of three subsamples, which were collected simultaneously and pooled to enhance microbial representation and ensure comprehensive coverage of the rhizosphere surrounding each date palm. Samples of the same cultivars were collected from trees located approximately 15 m apart from each other. The samples were taken from a depth of 20 cm within the root zone of 50‐year‐old date palm trees. Three soil samples were obtained from the 20 cm depth surrounding the root zone of each of three 50‐year‐old palm trees of each cultivar growing in close proximity. To maintain sample integrity, sterile equipment was used for soil collection, and the samples were immediately transferred to sterile plastic bags for transportation to the laboratory for subsequent DNA extraction and analysis.

### 2.2. DNA Extraction

Total DNA was extracted from 1 g of each soil sample using an UltraClean Soil DNA Purification Kit (Mo Bio Laboratories, Solana Beach, CA, United States) following the manufacturer′s protocol. The quantity and purity of the extracted DNA were assessed using a NanoDrop Spectrophotometer (Thermo Fisher Scientific).

### 2.3. PCR Amplification and Sequencing With the Illumina MiSeq Platform

Amplification of the V3–V4 hypervariable regions of the bacterial 16S rRNA gene was performed using the universal primer set 341F (5 ^′^‐CCTACGGGNGGCWGCAG‐3 ^′^) and 805R (5 ^′^‐GACTACHVGGGTATCTAATCC‐3 ^′^) [60, 61]. The amplification was outsourced to Macrogen (Seoul, Korea) and followed the Illumina 16S Metagenomic Sequencing Library protocols (https://www.illumina.com/). The PCR conditions consisted of an initial denaturation step at 95°C for 3 min, followed by 25 cycles of denaturation at 95°C for 30 s, annealing at 55°C for 30 s, and extension at 72°C for 30 s. A final extension step at 72°C for 5 min was included. Successful amplification was verified by gel electrophoresis using a 1% agarose gel stained with 0.5 *μ*g/mL ethidium bromide. After electrophoresis, the gel was visualized under a UV transilluminator to confirm the presence of the expected amplicon size (~450 bp). The purified amplicons were then submitted to Macrogen (Seoul, South Korea) for paired‐end sequencing on the Illumina MiSeq platform. Sequencing was performed on an Illumina MiSeq platform utilizing a 2 × 300 bp paired‐end read strategy. Raw 16S rRNA gene sequences were delivered in FASTQ format.

### 2.4. 16S rRNA Amplicon Bioinformatics Analyses

Following Illumina MiSeq sequencing, taxonomic assignment and downstream statistical analyses were performed on the raw 16S rRNA gene amplicon sequences using the EzBioCloud server 16S rRNA gene Microbiome Taxonomic Profiling (MTP) pipeline (https://www.ezbiocloud.net/contents/16smtp). Paired‐end reads were merged using VSEARCH v2.13.4 [62] within the MTP pipeline, followed by a quality filtering step to remove chimeric sequences, reads with low‐quality scores (*Q* < 25), and nontarget sequences. The remaining high‐quality reads were then clustered into operational taxonomic units (OTUs) at a 97% sequence similarity threshold using the UCLUST [63] algorithm implemented within the MTP pipeline, along with the EzBioCloud 16S database version PKSSU4.0 [64]. Alpha diversity indices, including Good′s coverage, rarefaction curves, observed OTUs, the Chao1 richness estimator (ACE), Simpson′s diversity index, and the Shannon diversity index, were calculated using the EzBioCloud MTP server. In EzBioCloud, statistical significance is generally assessed using the Wilcoxon rank‐sum test to compare *α* diversity indices, with a significance threshold of *p* < 0.05 [65].

To assess beta diversity, principal coordinate analysis (PCoA) based on the UniFrac distance metric was performed at the genus level by the EzBioCloud MTP server. A correlation network analysis was performed using DADA2 software after the eight samples were combined into only four samples—Ajwa, Helwa, Rabeaa, and Ruthana—based on the date palm cultivars used in the study [66]. A heat map depicting the distribution of identified PGPB across eight samples was generated using the SRplot online tool (https://www.bioinformatics.com.cn/en). A phylogenetic tree depicting the distribution of detected PGPB genera across the eight analyzed microbiomes was constructed using ClustalW for sequence alignment and the neighbor‐joining algorithm implemented in MEGA‐X software [67].

### 2.5. Predictive Functional Profiling and Phylogenetic Analysis

The functional prediction of PGP traits within the identified microbial communities was performed using the PICRUSt tool integrated within the EzBioCloud server. PICRUSt leverages the Kyoto Encyclopedia of Genes and Genomes (KEGG) Orthology database to predict functional profiles on the basis of 16S rRNA gene sequencing data. The tool employed the Kruskal–Wallis *H* test to identify differentially abundant functional biomarkers associated with PGPR activity at a significance level of *p* < 0.05 [68]. The resulting distribution of predicted PGP trait functional biomarkers was visualized using a heat map generated with the SRplot online tool (https://www.bioinformatics.com.cn/en).

### 2.6. Data Availability

The raw amplicon sequencing data generated in this study have been deposited into the National Center for Biotechnology Information (NCBI) Sequence Read Archive (SRA) database and are accessible under BioProject Accession Number PRJNA1158477.

## 3. Results

### 3.1. Characterization of the Date Palm Rhizosphere Bacterial Community Composition

To elucidate the bacterial community composition of date palm rhizospheres, 16S rRNA gene amplicon sequencing was performed on eight samples from four cultivars (Ajwa, Helwa, Rabeaa, and Ruthana). EzBioCloud MTP analysis yielded 569,596 valid reads, with a range of 65,329–84,860 per sample (Table 1).

**Table 1:** Alpha diversity indices (valid reads, percentage of valid reads, number of OTUs obtained, and Good′s library coverage [percentage]) assessed in the microbiomes of the date palm cultivars Ajwa, Rabeaa, Ruthana, and Helwa employed in this study.

Sample name	Valid reads	Valid reads percentage (%)	OTUs	Good′s coverage of the library (%)
AJW_I_1	84,860	98.1	1986	99.92
AJW_II_1	68,812	75.3	4414	99.11
HEL_I_1	66,995	73.6	4548	99.19
HEL_II_1	65,329	71.4	4429	98.92
RAB_I_1	71,976	79.4	3990	99.18
RAB_II_1	69,774	77.3	4141	99.17
ROT_I_1	72,237	78.6	4345	99.13
ROT_II_1	69,613	75.9	4664	99.19

**Figure 2:**
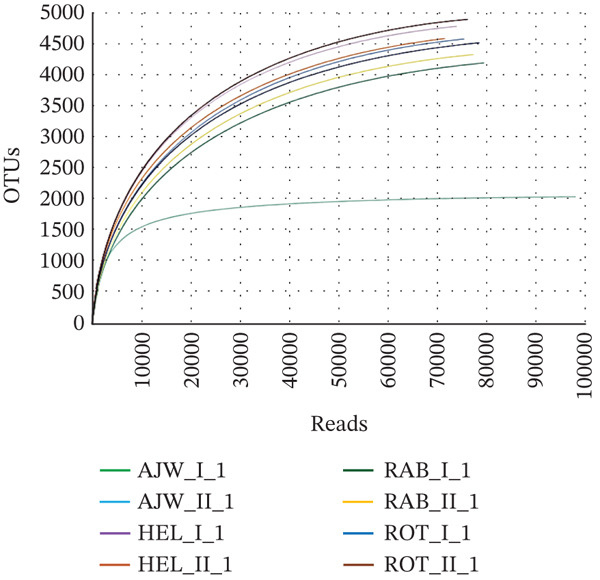
Rarefaction curves for the eight microbiomes identified in this study.

The percentage of valid reads ranged from 71.4% to 98.1% (Table 1). Rarefaction curves plateaued, and high Good′s coverage scores (> 99%) indicated sufficient sequencing depth to capture the bacterial diversity within the samples (Figure 2 and Table 1). These findings suggest that the sequencing data were representative of the rhizosphere microbiomes studied.

### 3.2. Diversity Analysis of Bacterial Communities

**Figure 3:**
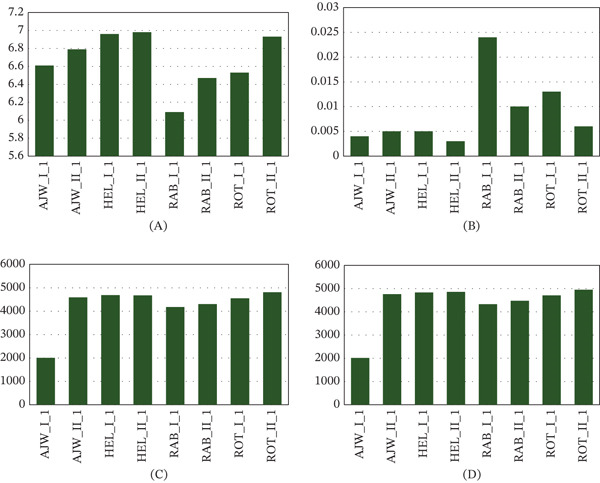
Alpha diversity indices for the microbiomes identified in this study: (A) the Shannon index, (B) the Simpson index, (C) the ACE index, and (D) the CHAO diversity index.

Alpha diversity indices (Shannon and Simpson indices) revealed significantly greater bacterial diversity in the AJW_I_1, AJW_II_1, HEL_I_1, HEL_II_1, and ROT_II_1 samples than in the RAB_I_1, RAB_II_1, and ROT_I_1 samples (Figure 3A,B). Additionally, the ACE and Chao1 indices (Figure 3C,D) indicated higher richness and evenness in the AJW_II_1, HEL_I_1, HEL_II_1, RAB_I_1, RAB_II_1, ROT_I_1, and ROT_II_1 samples than in the AJW_I_1 sample, which is consistent with the lower number of OTUs observed in AJW_I_1 (Table 1).

**Figure 4:**
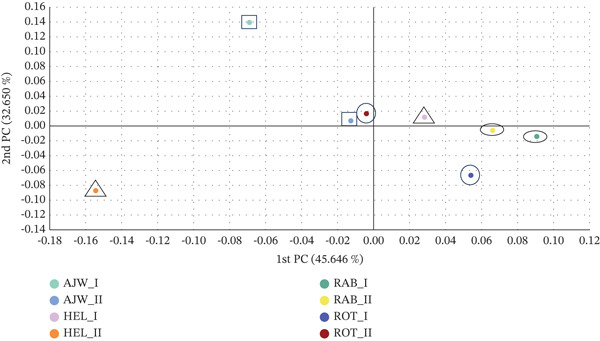
PCoA plot showing the beta‐diversity clustering of the eight microbiomes obtained in this study. Samples are grouped by cultivar using distinct markers: triangles (HEL), squares (AJW), circles (ROT), and ovals (RAB).

PCoA grouped the samples into four distinct clusters: AJW_1, HEL_II, ROT_1, and a combined cluster of the remaining samples (Figure 4). This suggests substantial variation in microbial composition, even among samples from the same date palm cultivar.

**Figure 5:**
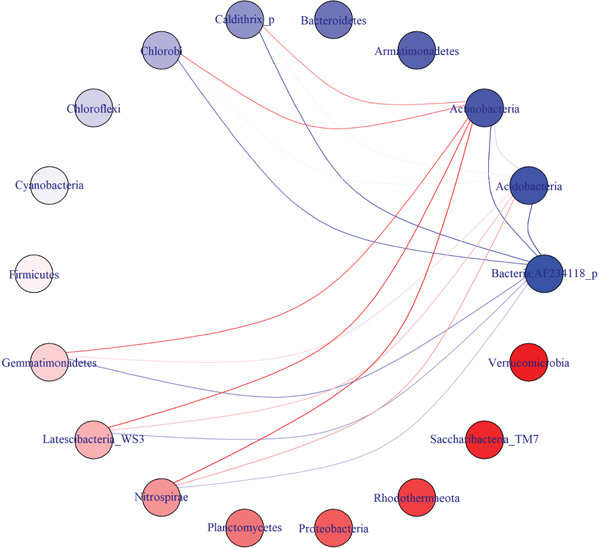
Analysis of correlations between OTUs (at the phylum level) using DADA2 software.

The highest positive correlations were clearly found between Caldithrixp and Bacteria af234111p and between Chlorobi and Bacteria af234111p (Figure 5).

### 3.3. Comparative Analysis of Date Palm Microbiomes

**Figure 6:**
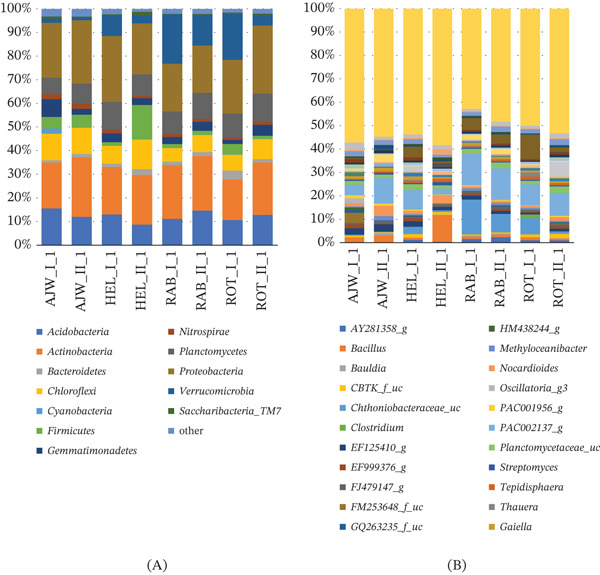
Relative abundances of bacteria identified in this study by phylum and genus. (A) Phylum and (B) genus.

Taxonomic classification of the raw sequences from the eight date palm rhizosphere samples was conducted using the EzBioCloud MTP server. At the phylum level (≥ 1% relative abundance), Proteobacteria dominated all the samples, except for RAB_I_1 and RAB_II_1, where the phylum Actinobacteria was most prevalent. Actinobacteria consistently ranked second, except in RAB_I_1 and RAB_II_1, where Verrucomicrobia and Proteobacteria were the second most abundant phyla, respectively (Figure 6A). This pattern suggests a similar distribution of the second and third most abundant phyla across the samples.

Acidobacteria, Chloroflexi, and Planctomycetes were less abundant but significantly represented in most samples.

At the genus level (≥ 1% relative abundance), the unclassified (PAC002137_g) genus from the order Actinomarinales consistently ranked second in all samples, except HEL_II_1 and RAB_I_1, where *Bacillus* and Chthoniobacteraceae_uc were the second most abundant genera, respectively. The remaining genera, whose relative abundances were less than 1%, collectively represented the largest proportion of the bacterial community in each sample (Figure 6B).

### 3.4. Distribution and Abundance of PGPB Genera

**Figure 7:**
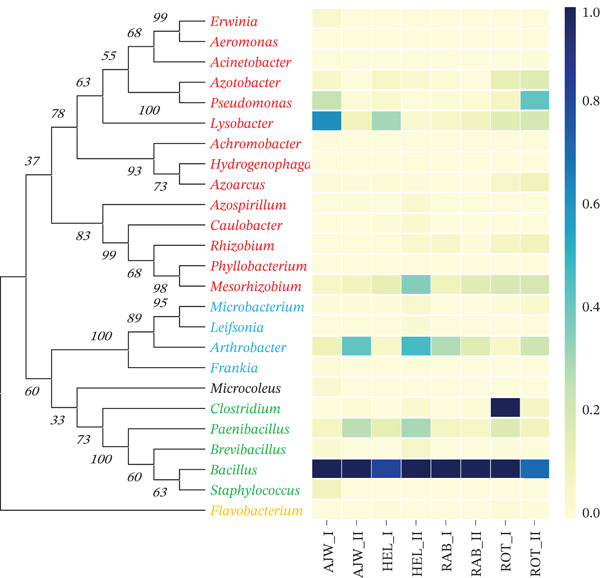
Clustered heat map showing the OHRB identified in the eight microbiomes obtained in this study. Genera from the phyla Proteobacteria, Actinobacteria, Cyanobacteria, Firmicutes, and Bacteroidetes are displayed in red, blue, black, green, and orange, respectively.

A clustered heat map illustrating the relative abundance of PGPB genera across our samples is presented in Figure 7. *Bacillus*, *Arthrobacter*, *Lysobacter*, *Paenibacillus*, and *Mesorhizobium* were consistently prevalent in most samples, with *Bacillus* being the most abundant. Genera such as *Clostridium*, *Rhizobium*, *Azoarcus*, *Azotobacter*, *Pseudomonas*, and *Erwinia* were more selectively represented, particularly in the ROT_I_1 and ROT_II_1 samples. *Aeromonas*, *Acinetobacter*, *Achromobacter*, *Hydrogenophaga*, *Phyllbacterium*, *Frankia*, and *Flavobacterium* were less abundant across most samples. Phylogenetic analysis of PGPB genera (Figure 7) revealed that *Microbacterium*, *Leifsonia*, *Arthrobacter*, and *Frankia* belonged to the phylum Actinobacteria; *Microcoleus* to Cyanobacteria; *Clostridium*, *Paenibacillus*, *Brevibacillus*, *Bacillus*, and *Staphylococcus* to Firmicutes; *Flavobacterium* to Bacteroidetes; and the remaining genera to Proteobacteria.

These findings demonstrate the widespread presence of PGPB genera in our samples and highlight the phylogenetic diversity within this group, with Proteobacteria being slightly overrepresented.

### 3.5. PICRUSt Identification of Potential Functional Genes for PGP Traits

**Figure 8:**
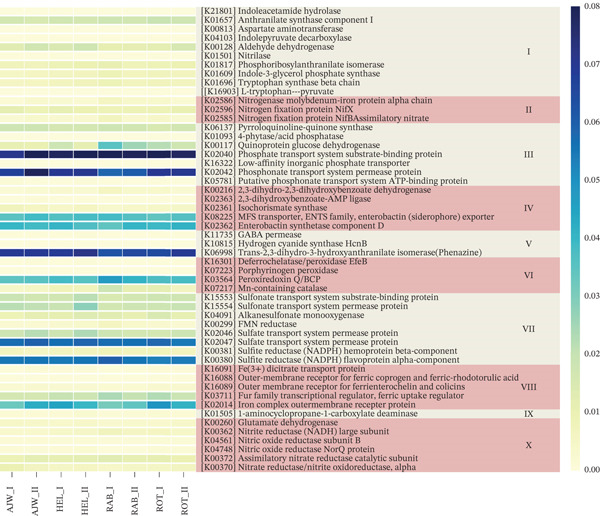
PICRUSt‐based heat map showing the major PGP traits identified using taxonomic 16S rRNA gene data, where I represents IAA production, II represents nitrogen fixation, III represents phosphate solubilization, IV represents siderophore production, V represents antimicrobial production, VI represents antioxidative stress, VII represents sulfur metabolism, VIII represents iron uptake, IX represents ACC deaminase, and X represents nitrogen metabolism.

By utilizing the taxonomic information from this study, PICRUSt analysis was employed to predict the functional potential of the microbial communities. As illustrated in Figure 8, PICRUSt analysis revealed the presence of genes associated with 10 PGP traits: indole acetic acid (IAA) production, nitrogen fixation, phosphate solubilization, siderophore production, antimicrobial production, oxidative stress resistance, sulfur metabolism, iron uptake, ACC deaminase, and nitrogen metabolism.

Among these PGP‐related genes, several exhibited notably higher relative abundances than others within the same trait or across different traits did. These genes included those encoding phosphate transport system substrate‐binding protein, phosphonate transport system permease protein, MFS transporter, ENTS family, enterobactin, enterobactin synthetase component D, trans‐2,3‐dihydro‐3‐hydroxyanthranilate isomerase, peroxiredoxin Q/BCP, sulfate transport system permease protein, sulfite reductase (NADPH) flavoprotein alpha‐component, and iron complex outer membrane receptor protein. The remaining potential PGP genes were identified at moderate (e.g., anthranilate synthase component I and pyrroloquinoline‐quinone synthase) to low (e.g., indoleacetamide hydrolase and aspartate aminotransferase) abundance levels (Figure 8). Collectively, these findings suggest that the PGP traits of the microbial communities studied, particularly those represented at high and moderate levels, play ecologically significant roles in the development of date palm trees in the sampling region. Furthermore, the data indicate a rich and potentially complex metabolic diversity concerning PGP traits within these samples.

## 4. Discussion

Date palm (*P. dactylifera*) is a commercially significant crop, particularly in arid regions such as the Middle East, and is valued for its food, traditional medicine, and nutritional content. PGPB are a diverse group of microorganisms that can directly increase plant growth through mechanisms such as nitrogen fixation, siderophore production, mineral solubilization, and phytohormone production. Additionally, PGPB can indirectly promote plant growth by inhibiting phytopathogens. PGPR, which colonize the rhizosphere, can stimulate plant growth and yield through both direct and indirect mechanisms. Direct mechanisms include facilitating nutrient uptake and improving mineral bioavailability [69–71]. Indirect mechanisms involve the production of several antimicrobial compounds, such as antibiotics and volatile organic compounds, such as hydrogen cyanide and hydrolytic enzymes [70, 72].

Extensive global research efforts have been directed toward uncovering beneficial microorganisms within diverse agroecological niches. In this study, 16S rRNA gene amplicon sequencing was used to characterize the bacterial community composition of date palm rhizosphere soils collected from Madinah, Saudi Arabia. Additionally, PICRUSt analysis was used to predict functional genes associated with various PGP traits on the basis of the 16S rRNA amplicon data. While culture‐independent metagenomic techniques have revolutionized the study of microbial diversity and abundance in rhizosphere soils, investigations focusing on the bacterial diversity of the date palm rhizosphere using these methods remain relatively scarce compared with those using culture‐dependent approaches [54]. Considering that the unified conditions were maintained during sampling, transportation, and sequencing (with Good′s coverage of the library exceeding 99%), some replicates did not cluster closely in the PCoA, which is likely due to biological factors associated with the host plant. Lei et al. supported this interpretation, demonstrating that plant species influence rhizosphere microbial community structure [73]. Moreover, since our samples were collected from trees approximately 15 m apart, the variability observed is consistent with the findings of Attia et al., who reported differences among small‐scale replicates from distinct root compartments and concluded that plant‐driven selection is the main determinant of root microbiome assembly, further highlighting the strong influence of plant‐associated factors on microbial community composition [74].

Proteobacteria and Actinobacteria were consistently the two dominant bacterial phyla in this study, while the other phyla were significantly less abundant. These findings align with those of Shamim et al., who reported the prevalence of Proteobacteria and Actinobacteria in date palm roots irrigated with both saline and nonsaline water [57]. Loganathachetti et al. also reported the dominance of Proteobacteria and Actinobacteria in soil under saline groundwater [75]. However, their study revealed a different pattern for nonsaline water irrigation, with Firmicutes and Actinobacteria being the most common phyla. A subsequent study by Loganathachetti et al. further confirmed the prevalence of Proteobacteria and Actinobacteria in date palm roots and Actinobacteria and Firmicutes in bulk soil under freshwater irrigation [76]. Al‐Busaidi et al. identified Proteobacteria and Actinobacteria as the overall predominant phyla in both normal and saline conditions of date palm rhizosphere soil, with Proteobacteria becoming more prevalent under saline conditions [77]. Additionally, Kouadri reported the presence of Actinobacteria, Proteobacteria, Firmicutes, and Bacteroidetes in date palm rhizosphere soil irrigated with groundwater and treated wastewater [78]. Collectively, these studies demonstrate the consistent dominance of Proteobacteria and Actinobacteria in date palm soil and rhizosphere environments.

A detailed analysis of the relative abundance of PGPB genera in the rhizosphere soil revealed *Bacillus*, *Arthrobacter*, *Lysobacter*, *Paenibacillus*, and *Mesorhizobium* as the most prevalent genera, with *Bacillus* being the most abundant. *Bacillus* spp., known for their widespread distribution in various soil types and their potential role in desert plant adaptation [79], exhibit high resistance to harsh environmental conditions. As a well‐established PGPB, *Bacillus* stimulates plant growth by increasing nutrient availability in the rhizosphere and inhibiting phytopathogens [80]. *Bacillus* has been associated with the production of phytohormones such as IAA and siderophores [81] and with phosphate solubilization. IAA, a key plant growth regulator (auxin), is a common metabolite of L‐tryptophan produced by several microorganisms, including PGPR. While soluble phosphorus and zinc are often deficient in natural soils, rhizospheric microorganisms can solubilize insoluble forms of these minerals, increasing their bioavailability in plants [82].

In addition to directly stimulating plant growth, PGPR can indirectly induce plant growth by suppressing soil‐borne pathogens. *Bacillus* is renowned for its ability to produce antibiotics such as zwittermicin A [83], bacilin, clorotetain, and iturin A [84]. Shamim et al. reported that *Bacillus*, *Micromonospora*, and *Mycobacterium* were the most prevalent PGPB in date palm root‐associated bacterial communities under saline water irrigation [57]. Compared with other PGPR, *Bacillus* spp. are particularly effective at controlling plant pathogens [85, 86]. Saha et al. demonstrated the antagonistic activity of two *Bacillus* strains against *Fusarium solani* through direct mechanisms involving fungal mycelium deformation [87]. *Bacillus* spp. have also been reported to control *Fusarium* wilt by degrading extracellular wall components [88]. Dual culture plate assays have further revealed significant antagonistic activity of *Bacillus subtilis* and *Bacillus* sp. against *Fusarium graminarium* and *Fusarium oxysporum*, respectively [89, 90].

Ferjani et al. employed 16S rRNA gene PCR‐DGGE to identify PGPB genera, including *Pseudomonas*, *Pantoea*, *Microbacterium*, *Bacillus*, *Arthrobacter*, *Enterobacter*, *Salinicola*, *Rhizobium*, and *Staphylococcus*, in date palm rhizosphere soil [55]. Yaish et al. characterized *Paenibacillus*, *Enterobacteras*, and *Bacillus* as endophytic PGPB and demonstrated their potential to promote date palm growth under saline stress through the production of ACC and IAA deaminases. IAA, a plant growth regulator, plays a crucial role in promoting lateral and adventitious root development, facilitating nutrient uptake, and enhancing plant growth [91].

*Enterobacter* genera are well recognized as valuable PGPR. Tilak et al. [92] and Ahemad and Kibret [93] emphasized the significance of nitrogen‐fixing and phosphate‐solubilizing PGPR, such as *Bacillus* and *Enterobacter*, for agricultural applications as soil inoculants to improve plant growth and yield. *Bacillus*, *Enterobacter*, and *Pseudomonas* isolated from arid soil have also been identified as potential PGPR that are capable of atmospheric nitrogen fixation, ammonia production, IAA production, siderophore production, and phosphate and zinc solubilization [94].

PICRUSt analysis revealed that more than 50 PGP genes are potentially involved in plant growth promotion, further supporting the importance of PGPB in date palm growth. This study represents the first report of the use of PICRUSt to predict PGP genes in the date palm rhizosphere. Among the PGP trait categories, genes encoding proteins for the phosphate transport system substrate‐binding protein and phosphonate transport system permease protein, which are crucial for phosphate transport and uptake, were consistently detected at high frequencies.

PGPR are well known for their ability to promote plant growth by converting inaccessible nutrient forms, such as nitrogen and phosphorus, into bioavailable forms that plants can absorb and utilize [69, 95]. Nitrogen‐fixing and phosphate‐solubilizing bacteria are particularly adept at converting atmospheric nitrogen into ammonia and hydrolysing insoluble organic phosphorus into soluble inorganic forms, respectively, thereby increasing the bioavailability of these essential nutrients for plant uptake.

PGPR are well recognized as biocontrol agents that effectively control plant diseases through various mechanisms, with antagonism being a key strategy [69]. The gene encoding trans‐2,3‐dihydro‐3‐hydroxyanthranilate isomerase, which is involved in phenazine biosynthesis, contributes to the inhibition of plant phytopathogens. Additionally, genes associated with iron uptake (e.g., quinoprotein glucose dehydrogenase), oxidative stress tolerance (e.g., methanol dehydrogenase), and siderophore production (e.g., isochorismate pyruvate lyase) were found to be abundant.

Tsurumaru et al. identified the abundance of genes encoding proteins involved in phosphate solubilization (e.g., quinoprotein glucose dehydrogenase), methanol utilization (e.g., methanol dehydrogenase), and siderophore production (e.g., isochorismate pyruvate lyase) during a metagenomic analysis of the bacterial community associated with the taproot of sugar beet [96]. Overall, microbiomes from the same cultivar tended to share similar dominant phyla. Proteobacteria were predominant in the date palm cultivars AJW, HEL, and ROT, whereas Actinobacteria dominated the RAB cultivar, indicating that the host cultivar influences microbiome composition at the phylum level. However, this influence was less consistent at the genus level; different genera dominated samples within the same cultivar in HEL and RAB, whereas a single genus was consistently dominant across samples in the other cultivars. The distribution of PGPB in our data revealed a generally similar pattern across cultivars, particularly for genera such as *Bacillus*, *Acinetobacter*, and *Lysobacter*. In contrast, certain genera, such as *Clostridium* and *Azoarcus*, were notably more abundant in samples from the ROT cultivar than in those from the other cultivars. Moreover, the distributions of genera such as *Pseudomonas* and *Arthrobacter* varied considerably, even between samples of the same cultivar. The distribution of functional biomarkers was generally consistent across the cultivars. However, in certain cases, such as quinoprotein glucose dehydrogenase (K00117), a notably higher abundance was observed in the RAB cultivar samples than in the others.

The primary objective of this study was to survey microbial composition and identify dominant taxa and general functional trends. To achieve this, we employed 16S rRNA gene amplicon sequencing as a well‐established and reliable approach for microbiome screening and community profiling. Functional insights were limited to predictive profiling inferred from taxonomic 16S rRNA amplicon data. While the number of biological replicates was limited, the results provide a representative overview of community structure and predicted metabolic potential, and future studies with expanded sampling and direct functional analyses are expected to provide additional support for the present findings.

## 5. Conclusion

Rhizobacteria isolated from desert plants demonstrate promising potential as biofertilizers because of their antagonism against pathogens and diverse PGP traits. These strains, which are adapted to harsh environments, could increase plant growth in desert regions. An analysis of date palm rhizosphere microbiomes from four cultivars (Ajwa, Helaw, Rabeaa, and Ruthana) collected in Madinah, Saudi Arabia, was conducted. The microbiome revealed significant variations in bacterial diversity and composition. Proteobacteria dominated, followed by Actinobacteria. PGPB genera were diverse, with *Bacillus*, *Arthrobacter*, *Lysobacter*, and *Paenibacillus* being predominant. The results of the phylogenetic analysis revealed broad representation across multiple phyla. PICRUSt analysis predicted the functional potential of the microbiome, highlighting PGP traits such as phosphate solubilization, siderophore production, oxidative stress resistance, and sulfur metabolism. These findings suggest the crucial role of the rhizosphere microbiome in date palm growth and health.

## Author Contributions

Each of the authors made equal contributions to conceptualizing and implementing the methodology, analyzing the data, and writing and revising the manuscript.

## Funding

This study was funded by Taibah University (10.13039/501100002403).

## Conflicts of Interest

The authors declare no conflicts of interest.

## Data Availability

The raw amplicon sequencing data generated in this study have been deposited into the National Center for Biotechnology Information (NCBI) Sequence Read Archive (SRA) database and are accessible under BioProject Accession Number PRJNA1158477.
